# Ethyl 4-[3-(2-methyl­benzo­yl)thio­ureido]benzoate

**DOI:** 10.1107/S160053680904183X

**Published:** 2009-10-17

**Authors:** Aamer Saeed, Hummera Rafique, Amara Mumtaz, Michael Bolte

**Affiliations:** a Department of Chemistry, Quaid-i-Azam University, Islamabad 45320, Pakistan.; b Institut für Anorganische Chemie, J. W. Goethe-Universität Frankfurt, Max-von-Laue-Str. 7, 60438 Frankfurt/Main, Germany.

## Abstract

The mol­ecular conformation of the title compound, C_18_H_18_N_2_O_3_S, is stabilized by an intra­molecular N—H⋯O hydrogen bond. The crystal packing shows centrosymmetric dimers connected by N—H⋯S hydrogen bonds. The terminal eth­oxy substituents are statistically disordered [occupancy ratio 0.527 (5):0.473 (5)].

## Related literature

For the use of thio­urea derivatives in organic synthesis and analysis, see: Eynde & Watte (2003 ▶); Fu *et al.* (1999 ▶); Rashdan *et al.* (2006 ▶); Maryanoff *et al.* (1986 ▶); Wang *et al.*(2005 ▶); Saeed *et al.* (2008 ▶); Koch, (2001 ▶). For their bioactivity and pharmaceutical applications, see: Upadhyaya & Srivastava (1982 ▶); Ramadas *et al.* (1998 ▶); Blum & Hayes (1979 ▶); DeBeer *et al.* (1936 ▶). For related structures, see: Saeed & Flörke (2007*a* ▶,*b* ▶); Saeed *et al.* (2009 ▶).
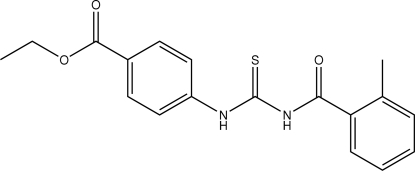


## Experimental

### 

#### Crystal data

C_18_H_18_N_2_O_3_S
*M* *_r_* = 342.40
Triclinic, 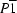

*a* = 7.4555 (3) Å
*b* = 7.6311 (4) Å
*c* = 15.2468 (8) Å
α = 96.456 (4)°
β = 103.860 (5)°
γ = 92.908 (4)°
*V* = 834.13 (7) Å^3^
*Z* = 2
Mo *K*α radiation
μ = 0.21 mm^−1^
*T* = 173 K
0.33 × 0.32 × 0.28 mm

#### Data collection

Stoe IPDS II two-circle-diffractometer
Absorption correction: multi-scan (*MULABS*; Spek, 2009 ▶; Blessing, 1995 ▶) *T*_min_ = 0.933, *T*_max_ = 0.943
22798 measured reflections
4659 independent reflections
4311 reflections with *I* > 2σ(*I*)
*R*_int_ = 0.057

#### Refinement

*R*[*F* ^2^ > 2σ(*F* ^2^)] = 0.037
*wR*(*F* ^2^) = 0.100
*S* = 1.04
4659 reflections
246 parameters
H-atom parameters constrained
Δρ_max_ = 0.28 e Å^−3^
Δρ_min_ = −0.37 e Å^−3^

### 

Data collection: *X-AREA* (Stoe & Cie, 2001 ▶); cell refinement: *X-AREA*; data reduction: *X-AREA*; program(s) used to solve structure: *SHELXS97* (Sheldrick, 2008 ▶); program(s) used to refine structure: *SHELXL97* (Sheldrick, 2008 ▶); molecular graphics: *XP* in *SHELXTL-Plus* (Sheldrick, 2008 ▶); software used to prepare material for publication: *SHELXL97*.

## Supplementary Material

Crystal structure: contains datablocks global, I. DOI: 10.1107/S160053680904183X/im2145sup1.cif

Structure factors: contains datablocks I. DOI: 10.1107/S160053680904183X/im2145Isup2.hkl

Additional supplementary materials:  crystallographic information; 3D view; checkCIF report

## Figures and Tables

**Table 1:** Hydrogen-bond geometry (Å, °)

*D*—H⋯*A*	*D*—H	H⋯*A*	*D*⋯*A*	*D*—H⋯*A*
N1—H1⋯O1	0.80	2.01	2.669 (1)	139
N2—H2⋯S1^i^	0.89	2.67	3.5551 (9)	170

Symmetry code: (i) 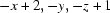.

**Fig. 1.**
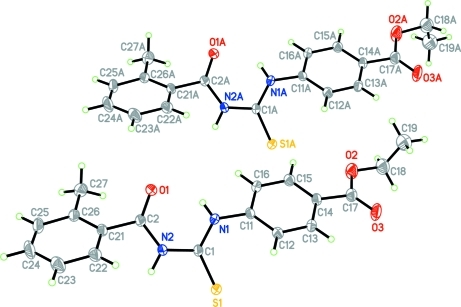
Perspective view of the title compound. The disordered atoms of the minor occupied site have been omitted for clarity. Displacement ellipsoids are shown at the 50 % probability level.

**Fig. 2.**
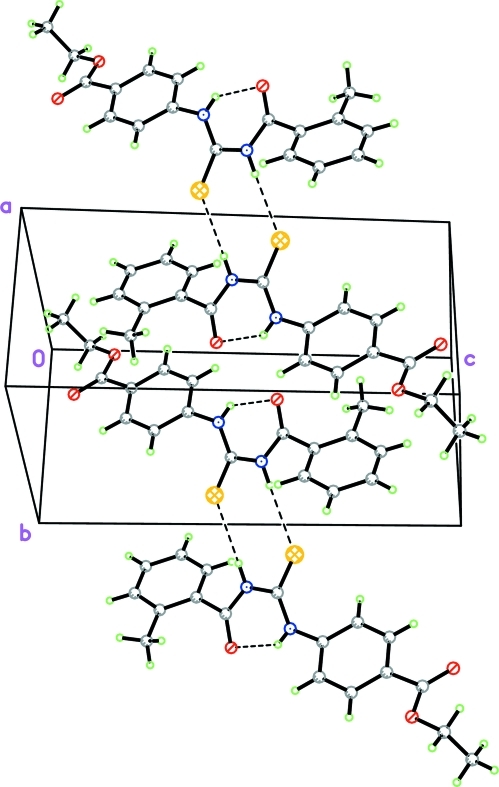
Packing diagram of the title compound. Hydrogen bonds shown as dashed lines. The minor occupied sites are omitted for clarity.

## supplementary crystallographic information

### Comment

The background of this study has been described in our earlier paper concerning the crystal structure of 1-(2-Chloro-5-nitrophenyl)-3-(2,2-dimethylpropionyl)thiourea (Saeed *et al.*, 2009). As part of our work on the structure of thioureas, we report here the structure of the title derivative, I, Fig 1.

The molecular conformation of the title compound, C_18_H_18_N_2_O_3_S, is stabilized by intramolecular N—H···O hydrogen bonds. The crystal packing shows centrosymmetric dimers connected by N—H···S hydrogen bonds (Table 1). Terminal ethoxy substituents are statistically disordered.

### Experimental

A solution of 2-methylbenzoyl chloride (10 mmol) in acetone (50 ml) was added dropwise to a suspension of potassium thiocyanate (10 mmol) in acetone (30 ml) and the reaction mixture was refluxed for 30 min. After cooling to room temperature, a solution of 4-aminobenzoic acid ethyl ester (10 mmol) in acetone (10 ml) was added and the resulting mixture refluxed for 3 h. The reaction mixture was poured into cold water and the precipitated thiourea was recrystallized from aqueous ethanol. Anal. calcd. for C_18_H_18_N_2_O_3_S: C, 63.14; H, 5.30; N, 8.18; S, 9.36% found: C, 63.26; H, 5.34; N, 8.21; S, 9.27%;

### Refinement

H atoms were positioned geometrically and refined using a riding model with fixed individual displacement parameters [U(H) = 1.2 *U*_eq_(C,N) or U(H) = 1.5 *U*_eq_(C_methyl_)] using a riding model with C—H(aromatic) = 0.95 Å, *C*—*H*(methyl) = 0.98 Å, or *C*—*H*(methylene) = 0.99 Å, respectively. H atoms bonded to N were set to the position where they were found in the difference map. The ethoxy group is disordered over two positions with a site occupation factor of 0.527 (5) for the major occupied site.

### Crystal data

**Table:**

C_18_H_18_N_2_O_3_S	*Z* = 2
*M**_r_* = 342.40	*F*(000) = 360
Triclinic, *P*1	*D*_x_ = 1.363 Mg m^−^^3^
Hall symbol: -P 1	Mo *K*α radiation, λ = 0.71073 Å
*a* = 7.4555 (3) Å	Cell parameters from 42517 reflections
*b* = 7.6311 (4) Å	θ = 3.4–29.9°
*c* = 15.2468 (8) Å	µ = 0.21 mm^−^^1^
α = 96.456 (4)°	*T* = 173 K
β = 103.860 (5)°	Block, colourless
γ = 92.908 (4)°	0.33 × 0.32 × 0.28 mm
*V* = 834.13 (7) Å^3^	

### Data collection

**Table:**

Stoe IPDS II two-circle-diffractometer	4659 independent reflections
Radiation source: fine-focus sealed tube	4311 reflections with *I* > 2σ(*I*)
graphite	*R*_int_ = 0.057
ω scans	θ_max_ = 29.6°, θ_min_ = 3.4°
Absorption correction: multi-scan (*MULABS*; Spek, 2009; Blessing, 1995)	*h* = −10→10
*T*_min_ = 0.933, *T*_max_ = 0.943	*k* = −10→10
22798 measured reflections	*l* = −21→21

### Refinement

**Table:**

Refinement on *F*^2^	Primary atom site location: structure-invariant direct methods
Least-squares matrix: full	Secondary atom site location: difference Fourier map
*R*[*F*^2^ > 2σ(*F*^2^)] = 0.037	Hydrogen site location: inferred from neighbouring sites
*wR*(*F*^2^) = 0.100	H-atom parameters constrained
*S* = 1.04	*w* = 1/[σ^2^(*F*_o_^2^) + (0.0517*P*)^2^ + 0.2098*P*] where *P* = (*F*_o_^2^ + 2*F*_c_^2^)/3
4659 reflections	(Δ/σ)_max_ = 0.002
246 parameters	Δρ_max_ = 0.28 e Å^−^^3^
0 restraints	Δρ_min_ = −0.37 e Å^−^^3^

### Special details

**Table:**

Geometry. All esds (except the esd in the dihedral angle between two l.s. planes) are estimated using the full covariance matrix. The cell esds are taken into account individually in the estimation of esds in distances, angles and torsion angles; correlations between esds in cell parameters are only used when they are defined by crystal symmetry. An approximate (isotropic) treatment of cell esds is used for estimating esds involving l.s. planes.
Refinement. Refinement of *F*^2^ against ALL reflections. The weighted *R*-factor *wR* and goodness of fit *S* are based on *F*^2^, conventional *R*-factors *R* are based on *F*, with *F* set to zero for negative *F*^2^. The threshold expression of *F*^2^ > σ(*F*^2^) is used only for calculating *R*-factors(gt) *etc*. and is not relevant to the choice of reflections for refinement. *R*-factors based on *F*^2^ are statistically about twice as large as those based on *F*, and *R*- factors based on ALL data will be even larger.

### Fractional atomic coordinates and isotropic or equivalent isotropic displacement parameters (Å^2^)

**Table:**

	*x*	*y*	*z*	*U*_iso_*/*U*_eq_	Occ. (<1)
S1	1.09603 (4)	0.22920 (4)	0.599200 (16)	0.02926 (8)	
O1	0.54635 (11)	0.37544 (11)	0.42973 (5)	0.03282 (18)	
O2	1.1748 (4)	1.1151 (2)	0.86115 (13)	0.0302 (5)	0.527 (5)
O2A	1.0912 (4)	1.1206 (2)	0.88161 (14)	0.0309 (6)	0.473 (5)
O3	1.23261 (17)	0.91072 (12)	0.96012 (6)	0.0521 (3)	
N1	0.82261 (12)	0.44800 (11)	0.57980 (5)	0.02358 (17)	
H1	0.7282	0.4728	0.5478	0.028*	
N2	0.77873 (12)	0.19546 (11)	0.47488 (6)	0.02420 (17)	
H2	0.8242	0.0956	0.4570	0.029*	
C1	0.89035 (13)	0.29850 (13)	0.55169 (6)	0.02227 (18)	
C2	0.61524 (13)	0.23496 (13)	0.41838 (6)	0.02368 (18)	
C11	0.90732 (12)	0.57062 (12)	0.65792 (6)	0.02124 (17)	
C12	0.98398 (14)	0.51635 (13)	0.74226 (7)	0.02457 (19)	
H12	0.9840	0.3941	0.7490	0.029*	
C13	1.06022 (14)	0.64342 (13)	0.81625 (7)	0.02587 (19)	
H13	1.1131	0.6075	0.8739	0.031*	
C14	1.06025 (15)	0.82288 (13)	0.80705 (7)	0.0273 (2)	
C15	0.97895 (15)	0.87601 (13)	0.72331 (7)	0.0286 (2)	
H15	0.9755	0.9983	0.7170	0.034*	
C16	0.90262 (14)	0.74958 (13)	0.64881 (6)	0.02458 (19)	
H16	0.8471	0.7856	0.5915	0.029*	
C17	1.1522 (2)	0.95152 (16)	0.88788 (9)	0.0456 (3)	
C18	1.2785 (4)	1.2514 (3)	0.93166 (16)	0.0336 (6)	0.527 (5)
H18A	1.3222	1.3504	0.9033	0.040*	0.527 (5)
H18B	1.3886	1.2024	0.9681	0.040*	0.527 (5)
C19	1.1602 (4)	1.3183 (4)	0.9924 (2)	0.0404 (6)	0.527 (5)
H19A	1.2319	1.4107	1.0394	0.061*	0.527 (5)
H19B	1.1193	1.2206	1.0215	0.061*	0.527 (5)
H19C	1.0518	1.3673	0.9564	0.061*	0.527 (5)
C18A	1.1603 (4)	1.2562 (4)	0.9587 (2)	0.0324 (6)	0.473 (5)
H18C	1.1639	1.2048	1.0158	0.039*	0.473 (5)
H18D	1.0751	1.3519	0.9553	0.039*	0.473 (5)
C19A	1.3507 (5)	1.3318 (4)	0.9602 (2)	0.0419 (8)	0.473 (5)
H19D	1.3942	1.4226	1.0131	0.063*	0.473 (5)
H19E	1.3469	1.3849	0.9043	0.063*	0.473 (5)
H19F	1.4356	1.2375	0.9644	0.063*	0.473 (5)
C21	0.53091 (13)	0.08983 (13)	0.34332 (7)	0.02406 (19)	
C22	0.50526 (15)	−0.08026 (14)	0.36571 (8)	0.0294 (2)	
H22	0.5425	−0.1013	0.4275	0.035*	
C23	0.42550 (17)	−0.21929 (16)	0.29831 (9)	0.0373 (3)	
H23	0.4064	−0.3349	0.3136	0.045*	
C24	0.37444 (18)	−0.18683 (18)	0.20872 (9)	0.0419 (3)	
H24	0.3212	−0.2812	0.1621	0.050*	
C25	0.40002 (17)	−0.01808 (17)	0.18616 (8)	0.0369 (3)	
H25	0.3647	0.0011	0.1241	0.044*	
C26	0.47678 (14)	0.12474 (15)	0.25293 (7)	0.0277 (2)	
C27	0.49622 (18)	0.30658 (17)	0.22573 (8)	0.0363 (2)	
H27A	0.3852	0.3678	0.2291	0.054*	
H27B	0.5109	0.2969	0.1632	0.054*	
H27C	0.6053	0.3737	0.2671	0.054*	

### Atomic displacement parameters (Å^2^)

**Table:**

	*U*^11^	*U*^22^	*U*^33^	*U*^12^	*U*^13^	*U*^23^
S1	0.02783 (13)	0.03461 (14)	0.02109 (12)	0.01339 (10)	−0.00154 (9)	−0.00282 (9)
O1	0.0292 (4)	0.0317 (4)	0.0296 (4)	0.0119 (3)	−0.0037 (3)	−0.0098 (3)
O2	0.0369 (11)	0.0226 (7)	0.0255 (8)	−0.0031 (6)	0.0009 (7)	−0.0036 (6)
O2A	0.0409 (14)	0.0227 (8)	0.0229 (8)	0.0024 (7)	−0.0005 (8)	−0.0048 (6)
O3	0.0761 (7)	0.0317 (4)	0.0293 (4)	−0.0019 (4)	−0.0204 (4)	−0.0010 (3)
N1	0.0232 (4)	0.0242 (4)	0.0187 (3)	0.0062 (3)	−0.0018 (3)	−0.0032 (3)
N2	0.0249 (4)	0.0245 (4)	0.0195 (4)	0.0070 (3)	0.0007 (3)	−0.0045 (3)
C1	0.0242 (4)	0.0250 (4)	0.0161 (4)	0.0044 (3)	0.0029 (3)	−0.0004 (3)
C2	0.0224 (4)	0.0264 (4)	0.0199 (4)	0.0046 (3)	0.0031 (3)	−0.0032 (3)
C11	0.0198 (4)	0.0235 (4)	0.0180 (4)	0.0019 (3)	0.0021 (3)	−0.0020 (3)
C12	0.0290 (4)	0.0217 (4)	0.0205 (4)	0.0023 (3)	0.0022 (3)	0.0007 (3)
C13	0.0305 (5)	0.0248 (4)	0.0188 (4)	0.0020 (4)	−0.0003 (3)	0.0017 (3)
C14	0.0304 (5)	0.0234 (4)	0.0219 (4)	−0.0016 (4)	−0.0035 (4)	−0.0004 (3)
C15	0.0337 (5)	0.0215 (4)	0.0252 (5)	−0.0009 (4)	−0.0020 (4)	0.0024 (3)
C16	0.0262 (4)	0.0253 (4)	0.0193 (4)	0.0023 (3)	0.0002 (3)	0.0025 (3)
C17	0.0634 (8)	0.0239 (5)	0.0322 (6)	−0.0038 (5)	−0.0185 (6)	−0.0001 (4)
C18	0.0361 (12)	0.0261 (11)	0.0317 (11)	−0.0045 (10)	0.0014 (9)	−0.0073 (9)
C19	0.0509 (14)	0.0331 (13)	0.0341 (14)	0.0081 (11)	0.0081 (11)	−0.0053 (11)
C18A	0.0436 (14)	0.0228 (12)	0.0262 (13)	0.0009 (10)	0.0060 (10)	−0.0101 (10)
C19A	0.0430 (15)	0.0352 (15)	0.0415 (15)	−0.0034 (13)	0.0032 (12)	−0.0028 (12)
C21	0.0200 (4)	0.0269 (4)	0.0222 (4)	0.0034 (3)	0.0035 (3)	−0.0065 (3)
C22	0.0272 (5)	0.0292 (5)	0.0310 (5)	0.0026 (4)	0.0085 (4)	−0.0031 (4)
C23	0.0334 (5)	0.0283 (5)	0.0479 (7)	−0.0032 (4)	0.0129 (5)	−0.0090 (5)
C24	0.0357 (6)	0.0405 (6)	0.0406 (6)	−0.0035 (5)	0.0054 (5)	−0.0208 (5)
C25	0.0342 (5)	0.0455 (6)	0.0244 (5)	0.0044 (5)	0.0018 (4)	−0.0121 (4)
C26	0.0243 (4)	0.0336 (5)	0.0222 (4)	0.0049 (4)	0.0036 (3)	−0.0053 (4)
C27	0.0398 (6)	0.0395 (6)	0.0283 (5)	0.0073 (5)	0.0056 (4)	0.0034 (4)

### Geometric parameters (Å, °)

**Table:**

S1—C1	1.6709 (10)	C18—H18A	0.9900
O1—C2	1.2247 (12)	C18—H18B	0.9900
O2—C17	1.370 (2)	C19—H19A	0.9800
O2—C18	1.451 (3)	C19—H19B	0.9800
O2A—C17	1.395 (2)	C19—H19C	0.9800
O2A—C18A	1.449 (4)	C18A—C19A	1.499 (4)
O3—C17	1.2038 (15)	C18A—H18C	0.9900
N1—C1	1.3388 (12)	C18A—H18D	0.9900
N1—C11	1.4220 (11)	C19A—H19D	0.9800
N1—H1	0.7998	C19A—H19E	0.9800
N2—C2	1.3824 (12)	C19A—H19F	0.9800
N2—C1	1.3936 (12)	C21—C22	1.3941 (15)
N2—H2	0.8911	C21—C26	1.4001 (14)
C2—C21	1.4937 (13)	C22—C23	1.3904 (15)
C11—C16	1.3895 (13)	C22—H22	0.9500
C11—C12	1.3941 (13)	C23—C24	1.382 (2)
C12—C13	1.3881 (13)	C23—H23	0.9500
C12—H12	0.9500	C24—C25	1.385 (2)
C13—C14	1.3923 (14)	C24—H24	0.9500
C13—H13	0.9500	C25—C26	1.4000 (14)
C14—C15	1.3900 (14)	C25—H25	0.9500
C14—C17	1.4858 (14)	C26—C27	1.5021 (17)
C15—C16	1.3904 (13)	C27—H27A	0.9800
C15—H15	0.9500	C27—H27B	0.9800
C16—H16	0.9500	C27—H27C	0.9800
C18—C19	1.493 (4)		
			
C17—O2—C18	115.67 (16)	C18—C19—H19A	109.5
C17—O2A—C18A	118.32 (17)	C18—C19—H19B	109.5
C1—N1—C11	126.33 (8)	H19A—C19—H19B	109.5
C1—N1—H1	116.3	C18—C19—H19C	109.5
C11—N1—H1	117.2	H19A—C19—H19C	109.5
C2—N2—C1	128.53 (8)	H19B—C19—H19C	109.5
C2—N2—H2	115.7	O2A—C18A—C19A	111.2 (4)
C1—N2—H2	115.5	O2A—C18A—H18C	109.4
N1—C1—N2	116.03 (8)	C19A—C18A—H18C	109.4
N1—C1—S1	125.74 (7)	O2A—C18A—H18D	109.4
N2—C1—S1	118.21 (7)	C19A—C18A—H18D	109.4
O1—C2—N2	122.79 (9)	H18C—C18A—H18D	108.0
O1—C2—C21	123.60 (9)	C18A—C19A—H19D	109.5
N2—C2—C21	113.61 (8)	C18A—C19A—H19E	109.5
C16—C11—C12	120.35 (8)	H19D—C19A—H19E	109.5
C16—C11—N1	117.45 (8)	C18A—C19A—H19F	109.5
C12—C11—N1	122.11 (9)	H19D—C19A—H19F	109.5
C13—C12—C11	119.08 (9)	H19E—C19A—H19F	109.5
C13—C12—H12	120.5	C22—C21—C26	120.98 (9)
C11—C12—H12	120.5	C22—C21—C2	118.37 (9)
C12—C13—C14	120.85 (9)	C26—C21—C2	120.65 (9)
C12—C13—H13	119.6	C23—C22—C21	120.47 (11)
C14—C13—H13	119.6	C23—C22—H22	119.8
C15—C14—C13	119.70 (9)	C21—C22—H22	119.8
C15—C14—C17	122.23 (10)	C24—C23—C22	119.01 (12)
C13—C14—C17	118.05 (9)	C24—C23—H23	120.5
C14—C15—C16	119.80 (9)	C22—C23—H23	120.5
C14—C15—H15	120.1	C23—C24—C25	120.70 (10)
C16—C15—H15	120.1	C23—C24—H24	119.7
C11—C16—C15	120.18 (9)	C25—C24—H24	119.7
C11—C16—H16	119.9	C24—C25—C26	121.39 (11)
C15—C16—H16	119.9	C24—C25—H25	119.3
O3—C17—O2	124.17 (12)	C26—C25—H25	119.3
O3—C17—O2A	120.61 (13)	C25—C26—C21	117.44 (11)
O3—C17—C14	124.28 (11)	C25—C26—C27	119.59 (10)
O2—C17—C14	109.38 (11)	C21—C26—C27	122.97 (9)
O2A—C17—C14	112.60 (11)	C26—C27—H27A	109.5
O2—C18—C19	110.5 (3)	C26—C27—H27B	109.5
O2—C18—H18A	109.6	H27A—C27—H27B	109.5
C19—C18—H18A	109.6	C26—C27—H27C	109.5
O2—C18—H18B	109.6	H27A—C27—H27C	109.5
C19—C18—H18B	109.6	H27B—C27—H27C	109.5
H18A—C18—H18B	108.1		
			
C11—N1—C1—N2	177.55 (9)	C15—C14—C17—O3	−175.89 (16)
C11—N1—C1—S1	−3.80 (15)	C13—C14—C17—O3	2.8 (2)
C2—N2—C1—N1	6.94 (15)	C15—C14—C17—O2	−12.1 (2)
C2—N2—C1—S1	−171.82 (9)	C13—C14—C17—O2	166.59 (17)
C1—N2—C2—O1	0.83 (17)	C15—C14—C17—O2A	22.0 (2)
C1—N2—C2—C21	−178.64 (9)	C13—C14—C17—O2A	−159.3 (2)
C1—N1—C11—C16	137.72 (11)	C17—O2—C18—C19	−77.4 (3)
C1—N1—C11—C12	−45.70 (15)	C17—O2A—C18A—C19A	79.8 (3)
C16—C11—C12—C13	−1.85 (15)	O1—C2—C21—C22	−128.25 (12)
N1—C11—C12—C13	−178.34 (9)	N2—C2—C21—C22	51.21 (12)
C11—C12—C13—C14	0.19 (16)	O1—C2—C21—C26	50.88 (15)
C12—C13—C14—C15	1.59 (17)	N2—C2—C21—C26	−129.66 (10)
C12—C13—C14—C17	−177.10 (12)	C26—C21—C22—C23	0.19 (16)
C13—C14—C15—C16	−1.71 (17)	C2—C21—C22—C23	179.32 (9)
C17—C14—C15—C16	176.93 (12)	C21—C22—C23—C24	0.89 (17)
C12—C11—C16—C15	1.74 (15)	C22—C23—C24—C25	−0.76 (19)
N1—C11—C16—C15	178.39 (9)	C23—C24—C25—C26	−0.44 (19)
C14—C15—C16—C11	0.06 (17)	C24—C25—C26—C21	1.48 (17)
C18—O2—C17—O3	−10.6 (4)	C24—C25—C26—C27	−177.99 (11)
C18—O2—C17—O2A	83.6 (3)	C22—C21—C26—C25	−1.35 (15)
C18—O2—C17—C14	−174.5 (2)	C2—C21—C26—C25	179.54 (9)
C18A—O2A—C17—O3	13.0 (4)	C22—C21—C26—C27	178.11 (10)
C18A—O2A—C17—O2	−93.5 (4)	C2—C21—C26—C27	−1.00 (15)
C18A—O2A—C17—C14	175.8 (3)		

### Hydrogen-bond geometry (Å, °)

**Table:**

*D*—H···*A*	*D*—H	H···*A*	*D*···*A*	*D*—H···*A*
N1—H1···O1	0.80	2.01	2.669 (1)	139
N2—H2···S1^i^	0.89	2.67	3.5551 (9)	170

Symmetry codes: (i) −*x*+2, −*y*, −*z*+1.
